# Utility and safety of airway stenting in airway stenosis after lung transplant: A systematic review

**DOI:** 10.3389/fmed.2023.1061447

**Published:** 2023-03-09

**Authors:** Zilin Wang, Bo Zhao, Mingming Deng, Run Tong, Yiding Bian, Qin Zhang, Gang Hou

**Affiliations:** ^1^Department of Pulmonary and Critical Care Medicine, Center of Respiratory Medicine, National Center for Respiratory Medicine, National Clinical Research Center for Respiratory Diseases, China-Japan Friendship Hospital, Beijing, China; ^2^Department of Pulmonary and Critical Care Medicine, Shengjing Hospital of China Medical University, Shenyang, China

**Keywords:** lung transplantation, airway, complications, stenosis, stents

## Abstract

**Background:**

Airway complications seriously affect the clinical outcomes and long-term prognosis of lung-transplantation patients. Airway stenting provides effective palliation for patients with airway stenosis. However, a lack of consensus regarding the efficacy and safety of airway stents in airway stenosis after lung transplantation. This study critically evaluated all available evidence regarding this concern.

**Methods:**

We retrieved studies from EMBASE, PubMed, and Cochrane Library databases. Studies were included if they reported baseline characteristics of airway complications after lung transplantation, stenting for airway stenosis, or prognosis.

**Results:**

In total, 279 papers were screened and 17 papers were included in final analysis. The short-term efficacy of airway stenting was assessed in almost all studies, with immediate palliation in symptom and improved pulmonary function reported. Eleven of the included studies evaluated the long-term efficacy of stent therapy, with no distinct lung function. The median overall survival time was 1,124 (95% confidence interval 415–1,833) days in stented patients only. Stent-related complications are common regardless of the material; However, serious complications are rare and can be improved with routine management.

**Conclusion:**

We demonstrated that airway stenting is a safe and effective method to treat airway stenosis after lung transplantation. The short-term effect was significant, while the long-term efficacy on survival rate needed further investigations.

**Systematic review registration:**

www.crd.york.ac.uk/prospero/, identifier: CRD42022364427.

## Introduction

Since the first lung transplantation in the 1960s, surgical techniques and medical support in this field have continuously improved. There are over 4,600 lung transplantations (LTx) performed worldwide annually, and the current median survival is 6.2 years (1–3). As lung transplantation techniques, donor preservation, and perioperative management have developed, the incidence of airway complications has declined (4–7). Airway complications after lung transplantation include stenosis, perioperative and postoperative bronchial infections, bronchial necrosis and dehiscence, excess granulation tissue, and tracheobronchomalacia (TBM) (8, 9), which remain major causes of morbidity and mortality after lung transplantation (10). Airway stenosis is the most common form (5) with endoscopic interventions such as cryoablation, laser photoresection, balloon bronchoplasty, electrocautery, brachytherapy, airway dilation using rigid bronchoscopy, and silicone or self-expanding metallic stent insertion, used for its management (11). With the update of materials and the maturity of endoscopic intervention technology, airway stent implantation remains a favorable option for improving airway stenosis (12). Although many studies have previously described the management of airway stenosis treated with stenting, only a few of studies on the efficacy and safety of different stenting procedures for airway stenosis after lung transplantation with only small subject numbers, a lack of consensus regarding the definitions of the subtypes of airway stenosis, and institution-specific bias (7). In addition, while some studies have identified the efficacy of stent insertion in solving airway stenosis after lung transplantation (13–15), others found no improvement in lung function in patients undergoing stent insertion after LTx (7, 16). Therefore, it is necessary to provide comprehensive evidence regarding this concern. Therefore, this study was designed to synthesize and critically evaluate all available evidence regarding the utility and safety of airway stenting in airway stenosis after lung transplantation, thus providing an overview of the evidence on the short- and long-term safety and efficacy of airway stenting in various studies, and to discuss the characteristics of patients who may benefit from the intervention.

## Methods and materials

The protocol for this systematic review was developed according to the Preferred Reporting Items for Systematic Reviews (PRISMA) checklist. This study did not require approval from the Institutional Review Board because no new data were collected, and all included studies had been published previously.

### Search strategy

Relevant studies were retrieved from the EMBASE, PubMed, and Cochrane Library databases from database inception through 12 September 2022. To identify the maximum number of relevant articles, a broad search strategy was employed, the research terms, including “lung transplantation,” “airway,” “stenosis,” “complication,” and “stents,” were searched in the combination of the Medical Subject Headings (MeSH) terms and keywords. The detailed search strategies are shown in Supplementary Data 1. The searches were not limited by date or publication status.

### Study selection

The inclusion criteria were any preclinical or clinical study in which (1) the patients included were adults (age ≥ 18 years); (2) the patients presented with airway stenosis after bilateral lung transplants or single lung transplant; (3) the patients received airway stent insertion; and (4) the follow-up results and outcomes were recorded. The exclusion criteria were: (1) incomplete or inaccurate studies or studies that failed to provide the outcome of airway stenting; (2) duplicate of already published literature; (3) bronchoscopic intervention without airway stenting; (4) studies not published in English; and (5) published in the form of letters, case reports, structured abstracts, or editorial reply. Selection procedure was presented in the PRISMA flow chart.

### Data extraction and conversion

Screening of the titles and abstracts of the retrieved studies for relevance was performed by two reviewers (BZ and ZLW), and a third reviewer (YDB) resolved disagreements arising from the inclusion process. Data extraction was performed independently by two reviewers (BZ and ZLW).

The data collected included: characteristics of each eligible studies, demographics of patients in each study including the age (median, range or median with or without standard deviation SD), primary diseases that led to lung transplantation of patients and their percentage, type of lung transplantation (single lung transplantation SLT, double lung transplantation DLT or heart lung transplantation HLT), airway complication types and so forth. The airway complication types would be recorded by the classification of bronchial stenosis, bronchomalacia, anastomotic dehiscence and granulation tissue. Cases with the phenotypes of bronchial stenosis would also be extracted, either the location-based (central airway strictures CAS and distal airway strictures DAS) or bronchoscopy-appearance-based classification (cicatrix scar, pseudomembrane, granulation tissue). For the evaluation of efficacy of stenting, we sought the follow-up period, follow-up outcomes and their changes before and after insertion of stents, including the symptoms, pulmonary function test (FEV_1_, FVC), complication incidence, survival or mortality rate, airway patency, or number of bronchoscopies; data of post-stent complications were extracted for the evaluation of safety of stenting. We also extracted the limitations of each included study to critically interpret the evidence if they were available. No meta-analyses were performed due to the heterogeneity of the included studies. Therefore, we tabulated the extracted data for the presentation of results.

For data that could not be directly extracted such as the age, the complication rates of stenting or the percentage of primary diseases, we reported the median and range age of the sample for age, calculating the rate of which patients with post-stent complications accounted in all lung-transplantation patients with stent insertion for complications rate. For the percentage of primary diseases, we calculated proportion of patients with primary diseases in the whole stented LTx patients. Data that cannot be found throughout article or cannot be converted through other data would be presented as NA for the meaning of “not available.” For the high heterogeneity among the included studies, only those with exact survival time of death were extracted to synthesize overall survival time. Software (IBM SPSS Statistics for MacOS, Version 26.0. Armonk, NY: IBM Corp) was used and Kaplan-Meier method was conducted for survival analysis.

### Quality assessment

The study quality was assessed using The Joanna Briggs Institute (JBI) Critical Appraisal Checklist for cohort study and the JBI Critical Appraisal Checklist for case series (17). According to JBI checklist, cohort studies were classified as follows: high quality (8–11 “Yes” response), moderate quality (4–7 “Yes” response), low quality (1–3 “Yes” response). Case series studies were considered high quality (7–8 “Yes” response), moderate quality (4–6 “Yes” response), low quality (1–3 “Yes” response) of methodology.

## Results

### Characteristics of the included studies

**Figure 1:**
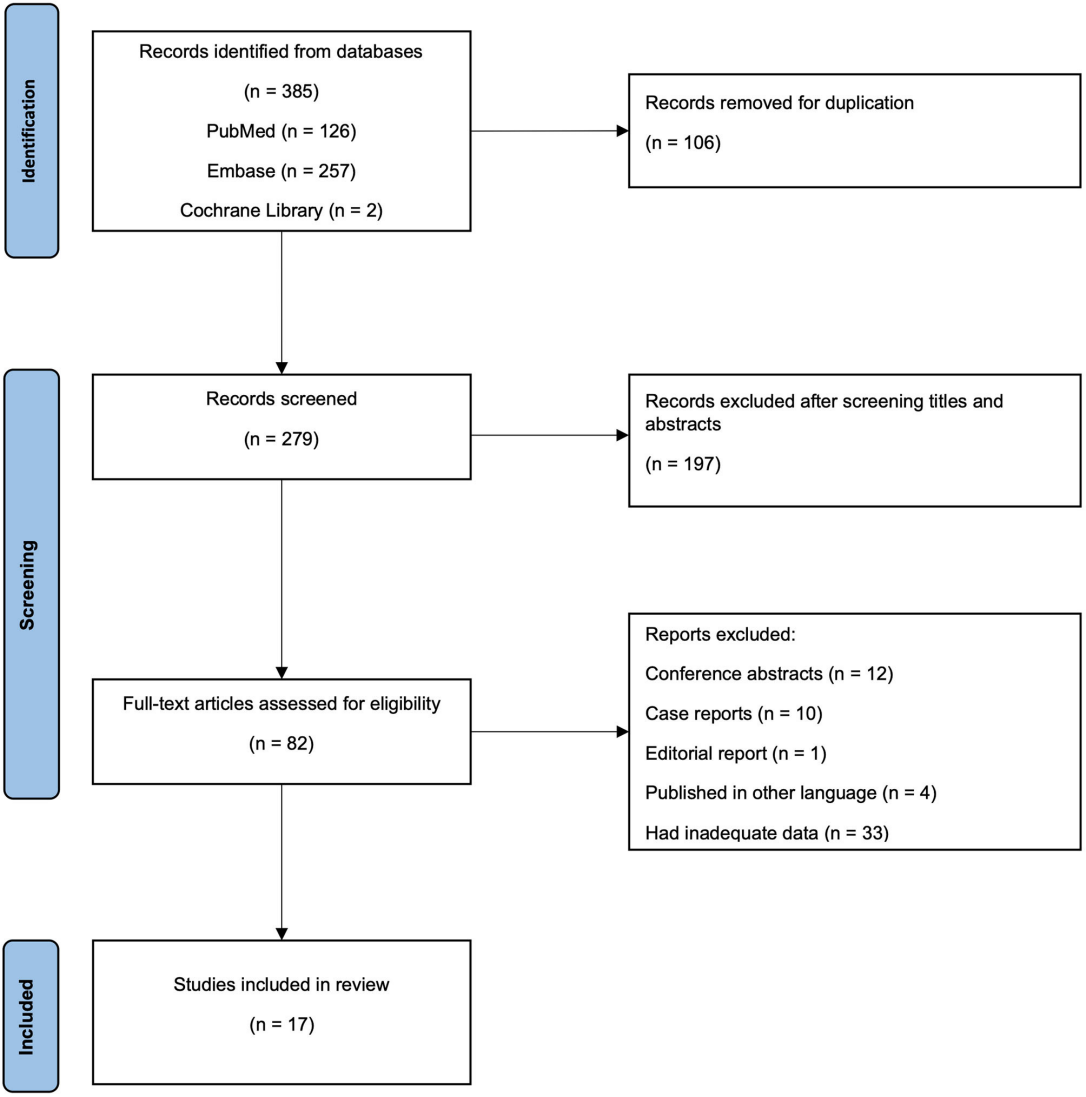
Preferred reporting items for systematic reviews checklist flow diagram. A total of 385 potentially relevant records were retrived from three databases. One hundred six records were excluded due to duplications. The remaining 279 records were then scrrened through titles and abstracts for relevance and 197 records were removed, leaving 82 records to be assessed through full text. Finally, there were 17 records considered to be included for further analysis.

This study identified 279 potentially relevant studies. 82 studies were considered to be relevant; however, 65 were excluded due to being conference abstracts, case reports, editorial report, including inadequate data, such as the data reported by Kshettry et al. (18), the exact patients who eventually were implanted with stents were unclear, so that the demographics of patients could not be extracted. Seventeen studies were found to be eligible and met all the inclusion criteria (3, 7, 13–16, 19–29). Figure 1 shows the selection procedure and outcomes of the literature search. The data were mostly published after 2000 (15/17, 88.2%), and the duration of follow-up was ~1 year or more. All 17 studies were retrospective, with a relatively small sample size, ranging from four to 65 patients. A total of 410 (median 20) patients experienced airway complications that needed the insertion of stents after lung transplantation. Three of included studies used stents for the resolution to not only the stricture duo to bronchial stenosis and bronchomalacia, but anastomotic dehiscence as well (3, 21, 23). Two studies reported the endobronchial phenotypes of bronchial stenosis as cicatrix scar, pseudomembrane and granulation tissue (7, 21). Seven studies recorded the stenosis according to the location of stenosis as CAS or DAS (7, 14–16, 20, 25, 26). Eleven studies used metallic stents to treat stenosis (3, 14–16, 19, 23–25, 27–29), three implanted silicone stents (11, 20, 26), two studies used both (7, 21). Another study used novel biodegradable stents (13). The population mainly comprised young and middle-aged adults. More details on the characteristics of the included studies and their patients are shown in Tables 1, 2.

**Table 1:** Characteristics of studies.

**Study**	**References**	**Span of time**	**Stent material**	**Suture method**
1	DiBardino et al. (7)	Between July 2012 and July 2018.	Uncovered metal stent (UCMS), Covered metal stent (CMS), Silicone stent.	NA
2	Kapoor et al. (19)	From 1992 to 2003.	Self-expanding, non-covered metallic stents.	NA
3	Thistlethwaite et al. (20)	Between January of 1996 and December of 2007.	Silicone stent.	End-to-end technique using a continuous nonabsorbable monofilament (3–0 polypropylene) suture
4	Ma et al. (21)	Between October 2012 and October 2017.	UCMS, CMS, Silicone stent.	NA
5	Dutau et al. (11)	Between January 1997 and December 2007.	Silicone stent.	NA
6	Fernandez-Bussy et al. (23)	Between February 2007 and April 2008.	A fully covered, self-expanding, hybrid nitinol stent.	End-to-end anastomoses with continuous sutures.
7	Izhakian et al. (24)	Between January 2002 and January 2018.	Self-expandable metal stent (61 SMART and 2 PALMAZ)	NA
8	Abdel-Rahman et al. (25)	Between January 1997 and March 2013	Self-expanding metal stent	Absorbable suture material polydioxanone (PDS, Ethicon, Inc., NJ, USA) was used. A continuous suture of the membranous wall (PDS, 4/0) and end-to-end anastomosis with interrupted single sutures (PDS, 3/0) of the cartilaginous part was performed. The first suture to unite the cartilaginous parts was placed in the middle of the circumference to achieve optimal size matching. In none of the patients, a viable tissue patch was used.
9	Saad et al. (3)	Between January 1992 and November 2001.	Self-expandable metallic stent (10 WS and 5 UF)	NA
10	Gottlieb et al. (16)	Between January 1998 and February 2008.	Self-expanding metal stent (91% uncovered and 9% covered)	NA
11	Sundset et al. (26)	Between 1990 and 2008.	Silicone stents (Hood or Dumont type)	Reconstruction of the airway was performed with continuous polypropylene stitch until 1996. Thereafter interrupted stitches on the cartilaginous part and running polydioxanone (PDS) stitches on the membranous part were used. Soft tissues were respected and anastomosis was performed on the cartilaginous ring proximal to the upper lobe bronchus or above the tracheal carina. An intercostal bundle wrap was used initially on unilateral procedures but was later abandoned. No bronchial artery revascularization was performed.
12	Susanto et al. (27)	Between December 1988 and January 1997	Balloon-expandable metallic stent	All patients underwent the same surgical technique regarding the bronchial anastomosis
13	Lischke et al. (13)	Between 2006 and 2010.	Novel biodegradable stents.	The absorbable suture material PDS (Ethicon Inc., NJ, USA) was used. A continuous suture of the membranous wall (PDS, 4/0) and end-to-end anastomosis with interrupted single sutures (PDS, 4/0) of the cartilaginous part were performed. No telescoping was used.
14	Burns et al. (14)	Between February 1996 and April 1999.	Self-expanding metal stents.	NA
15	Bolot et al. (15)	Between August 1991 and October 1995.	Self-expanding metallic stents.	NA
16	Anile et al. (28)	NA	Self-expandable metallic stents	NA
17	Fonseca et al. (29)	Between August 2003 and April 2014	Self-expansible metallic stents made of Nitinol	Continuous sutures in the membranous wall and separate stitches in the anterolateral wall of the bronchi with the use of 4–0 nonabsorbable monofilament polypropylene thread

NA, not available.

**Table 2:** Patient demographics.

**Study**	**No. stented patients**	**Gender (M)**	**Gender (F)**	**Age (year)**	**Primary diseases (%)**	**Days between transplant and airway complication first identified**	**Location of stent (%)**	**LTx procedure (%)**
1	4	6 (66.7%)	3 (33.3%)	55 ± 12	ILD/IPF 5 (56)	52 (40–61) days	Right upper lobe 9 (32.1)	DLT 8 (89)
					Pulmonary hypertension 2 (22)		Bronchus intermedius 6 (21.4)	SLT 1 (11)
					COPD 1 (11)		Right middle lobe 3 (10.7)	
					CF 1 (11)		Right lower lobe 2 (7.1)	
2	25	16 (64%)	9 (36%)	Average 51.6	NA	NA	NA	NA
				Range (21–65)				
3	20	15 (15/22)	7 (7/22)	44.2 ± 13.3	COPD 8 (40)	Average 81.5 ± 26.9	Right lung 11 (50)	DLT11 (55)
					CF 6 (30)	Range (35–135) das	Others 11 (50)	SLT 9 (45)
					IPF 3 (15)			
					Bronchiectasis 3 (15)			
					Lymphangiomyomatosis 1 (5)			
					Eosinophilic granulomatosis 1 (5)			
4	36	22 (61.1%)	14 (38.9%)	60.53 ± 9.68	COPD 10 (27.8)	127 (82–201) days	Right mainstem stenosis 10 (21.3)	NA
					IPF/ILD 23 (63.9)		Left mainstem stenosis 22 (46.8)	
					CF 1 (2.8)		Bronchus intermedius 11 (23.4)	
					COPD and IPF/ILD 1 (2.8)		Peripheral 4 (8.5)	
					Other 1 (2.8)			
5	17	10 (58.8%)	7 (41.2%)	37 ± 15	CF 11 (64.7)	165 (5–360) days	Right lung 9 (52.9)	DLT14 (82.4)
					Emphysema 3 (17.6)		Left lung 8 (47.0)	SLT 3 (17.6)
					Bronchiectasis 1 (5.9)			
					Sarcoidosis 1 (5.9)			
					Bronchioloalveolar carcinoma 1 (5.6)			
6	24	15 (62.5%)	9 (37.5%)	Average 60	COPD 9 (37.5)	1–26 months after lung transplantation	Bronchus intermedius 10 (20.4)	DLT 12 (50)
				Range (44–68)	Pulmonary fibrosis 7 (29.2)		Left anastomosis 19 (20.4)	SLT 12 (50)
					Bronchiolitis obliterans syndrome 5 (20.8)		Right anastomosis 19 (20.4)	
					Sarcoidosis 2 (8.3)		Left lower lobe 1 (2)	
					Lymphangioleiomyomatosis 1 (4.2)			
7	54	30 (55.5%)	24 (44.5%)	52.9 ± 12.0	Pulmonary fibrosis 18 (33.3)	Median 163.5 days	Right main bronchus 30 (47.6)	DLT18 (33.3)
					Emphysema 17 (31.5)	Range (12–2,134) days	Left main bronchus 33 (52.4)	SLT36 (66.7)
					CF 6 (11.1)			
					Bronchiectasis 6 (11.1)			
					Others 7 (13)			
8	47	NA	NA	NA	CF 5 (10.6)	Median159 days	Right main stem bronchus 28 (46.7)	NA
					Emphysema 16 (34)	Range (15–2160) days	Left main stem bronchus 30 (50)	
					Pulmonary fibrosis 12 (25.5)		Left upper lobe bronchus 1 (1.7)	
					Pulmonary hypertension 2 (4.3)		Lingular bronchus 1 (1.7)	
					Others 12 (25.5)			
9	12		6 (50%)	47.3 ± 9.6	IPF 4 (33.3)	Average 20.1 ± 19.5 months	Bronchus intermedius 2 (16.7)	DLT 6 (50)
					CF 3 (25)	Range (1.2–58) months	Right main bronchus 3 (25)	SLT 6 (50)
					COPD 2 (16.7)		Left main bronchus 8 (66.7)	
					Alpha1-antitrypsin deficiency 2 (16.7)			
10	65	40 (65%)	25 (35%)	48 (17–64)	Emphysema 30 (46)	133 (55–903) days	Intermediate bronchus 59 (53.2)	DLT 61 (94)
					Cystic fibrosis 13 (20)		Right upper lobe bronchus 11 (10)	SLT 2 ()
					Pulmonary fibrosis 13 (20)		Right lower lobe bronchus 1 (0.9)	
					Pulmonary artery hypertension 1 (8.3)		Middle lobe bronchus 1 (0.9)	
					Other 8 (12)		Left main bronchus 16 (14.4)	
							Left upper lobe bronchus 12 (10.8)	
							Left lower lobe bronchus 11 (10)	
11	27	16 (59.3%)	11 (40.3%)	Median 53	NA	Median 97 days	Trachea 1 (3.1)	DLT 19 (70.4)
				Range (21–61)		Range (32–243) days	Right main bronchus 5 (15.6)	SLT 7 (25.)
							Right bronchus intermedius 7 (21.9)	
							Left main bronchus 19 (59.4)	
12	6	0	6 (100)	46 ± 11	IPF 4 (66.7)	NA	NA	SLT 5 (83.3)
					COPD 1 (16.7)			BLT 1 (16.7)
					PPH 1 (16.7)			
13	6	3 (50%)	3 (50%)	Median 41.5	IPF 1 (16.7)	NA	NA	DLT 4 (66.7)
				Range (35–57)	CF 1 (16.7)			SLT 2 (33.3)
					COPD 4 (66.7)			
14	30	11 (36.7%)	19 (63.3%)	39.2 ± 11.7	Emphysema 8 (25)	Median 182.5 days	NA	NA
					IPF 6 (18.8)	Range (23–341) days		
					Sarcoidosis 2 (6.3)			
					Eisenmengers 7 (21.9)			
					PPH 2 (6.3)			
					OB 2 (6.3)			
					Cystic fibrosis 5 (15.6)			
15	18	5 (27.8%)	13 (72.2%)	45 (25–64)	Emphysema 7 (38.9)	5.6 (1–57) months	NA	DLT 5 (27.8)
					Cystic fibrosis 1 (5.6)			SLT 13 (72.2)
					primary pulmonary hypertension 6 (33.3)			
					IPF 2 (11.1)			
					Histiocytosis X l (5.6)			
					Bronchioloalveolar carcinoma 1 (5.6)			
16	10	NA	NA	25.2 ± 13.1	CF6 (60)	NA	Right main bronchus 8 (72.8)	SLT 2 (20)
					IPF 2 (20)		Bronchus intermedius 3 (27.3)	BLT 8 (80)
					Bronchiectasis1 (10)			
					Emphysema 1 (10)			
17	9	6 (66.7)	3 (33.3)	Median 46	Pulmonary microlithiasis 1 (11.1)	Median 7	Right anastomosis 3	SLT 2 (22.2)
				Range (20–58)	Bronchiectasy 2 (22.2)	Range (2–15) months	Left anastomosis 8	BLT 7 (77.8)
					Silicosis 1 (11.1)			
					Bronchiolitis obliterans 1 (11.1)			
					COPD 2 (22.2)			
					CF 1 (11.1)			
					IPF 1 (11.1)			

No., number; COPD, chronic obstructive pulmonary disease; IPF, idiopathic pulmonary fibrosis; ILD, interstitial lung disease; OB, bronchiolitis obliterans; PPH, primary pulmonary hypertension; SLT, single lung transplantation; DLT, double lung transplantation; HLT, heart lung transplantation; NA, not available.

### Efficacy and safety of the included studies

All 17 studies focused on the efficacy and safety of stent therapy. Efficacy was evaluated by the improvement of symptoms, lung function, change in the number of bronchoscopies, survival time, degree of stenosis, and infection rate, while safety was assessed by the occurrence and severity of post-stenting complications. The details of the contents and results of these studies are presented in Table 3.

**Table 3:** Stents-related contents and clinical results.

**Study**	**Number of stents used**	**Indications of stent-planting (*n*)**	**Right bronchial system complications (*n*)**	**Stent complications**	**Complication rate**	**Clinical results**
1	36	All DAS	Right upper lobe 2	Migration	80% (4/5 patients)	No improvement in lung function
			Right middle lobe 1	Granulation tissue		No reduction in bronchoscopies per month
			Right lower lobe 1	Mucous plugging		No difference in airway patency for stented patients
			Bronchus intermedius 2			
2	27	BM 9	Right main stem bronchus 3	Granulation tissue migration	4.9% (per patient per mont)	84% of the patients experienced immediate relief from dyspnea.
		BS 12	Right main stem bronchus 6	Infection hemoptysis		The mean percentage change in FEV_1_ was significantly greater than zero at 1 and 6 months (*P* < 0.05) post-stent placement.
		AD 3	NA	Collapse		FVC is marginally improved for 6 months (*P* > 0.05).
						Changes of FEV_1_ are marginal and may return to base line at 12 months
3	NA	CAS 19	Right anastomosis 8	Granulation tissue	NA	A significant increase in FEV_1_, FVC and FEV_1_/FVC ratio after therapy.
		DAS 3	Right anastomosis 3	Stent fracture with wires protrusion		Airway patency and symptom improvement were achieved in 18 of 20 patients.
				Stent erosion through the airway		16 of 20 patients were able to have their stents removed at a mean of 362.3 ± 126.4 days with permanent resolution of airway stenosis.
				Longitudinal stent collapse		Overall survival was similar for patients with and without airway stenosis.
				Stent removal		
4	47	AD 6	NA	Mucous plugging	NA	Improvement in FEV_1_ after stent placement was only observed in patients who ultimately were able to undergo stent removal.
		Cicatrix 19		Granulation tissue		A statistically significant reduction in number of bronchoscopies per month after stent removal compared to pre-stent placement.
		Pseudomembrane 14				
		BM 19				
5	NA	BS 18	Right anastomosis 7	Obstructive granulomas	13% (per patient per month)	Symptomatic improvement was noted in all patients, and FEV_1_ increased significantly after stent insertion.
		BM 2	Right anastomosis 1	Mucus plugging		Definitive stent removal in 12 of 17 (70.6%) patients without recurrence
		BS+BM 3	Right anastomosis 1	Migration		Overall survival was similar
6	49	BS 12	Right anastomosis 4	Granulation tissue	Similar to those previously reported for silicone and metal stent.	The average degree of stenosis decreased from 80 to 20%.
		AD 5	Right anastomosis 4	Migration		FEV_1_ and FVC increased significantly after stent insertion.
		BM 12	Right anastomosis 6	Stent fracture		
		BM+BS 20	Right anastomosis 5	Thick mucus formation		
7	63	BS 63	NA	Severe purulent secretion Endobronchial bleeding Unscheduled bronchoscopy	A	A significant increase in the mean FEV_1_ 60 days after stent insertion in the intervention group.
				Stent collapse Stent pressure ulcer Polyps in stent Stent fractures		The FEV_1_ values were significantly lower in the intervention than in the control group at 2.5 years of post-transplantation
				Stent malposition Migration Necrosis		The mean FEV_1_ values did not differ significantly between the intervention and control groups at 5, 7.5, and 10 years.
8	60	CAS 58	Right anastomosis 28	Granulation tissue Restenosis	NA	Provide immediate improvement in symptoms and pulmonary function tests in most cases.
		DAS 2		Fever		Mortality is similar to patients without stent insertion.
9	15	BS 7	Right main bronchus 3	Bacterial bronchitis	4% (per patient per month)	A non-statistically significant trend toward improvement was observed in FEV_1_.
		AD 2	Bronchus intermedius 1 Right main bronchus 1	Obstructive granulomas		The overall 5-year survival rate for patients with SEMSs was similar compared with others.
		BM 6	Bronchus intermedius 1			Stent insertion before day 90 proved to be an independent covariate associated with re-stenosis.
10	111	BS*	Overall right bronchus 72	Mucus plugging.	80%	27 (41.5%) patients developed BOS at 853 (104–1848) days after stent insertion.
		AD*		Restenosis		Overall survival was significantly lower in recipients with SEMS than the total cohort
				Bacterial colonization		BOS-free survival was not affected.
				Hemoptysis		
				Stent fracture		
				Atelectasis		
11	32	BS 32	Right main bronchus 5	Mucous plugging	Common	Symptoms were relieved and FEV_1_ increased in all patients.
			Bronchus intermedius 7	Granulation tissue		25 stents in 21 patients were removed after 6 months (median). 22 of the stented airways remained patent and lung function were stable during the 24 months.
				Migration		The mean percentage change in FEV_1_ was 43 ± 44% (−30 to +100)
12	10	BS 2	Right main bronchus 2	Stent dehiscence	Rare	The mean percentage change in FVC was 17 ± 24% (−15 to +57)
		BM 2	Bronchus intermedius 1 Right lower lobe 1	Stent collapse		
		BS+BM 2	Right main bronchus 1			
13	20	CAS 9	NA	Perianastomotic necrosis of the bronchus	NA	The stenosis was initially relieved in all cases.
		DAS 7		Stenosis		There was no bleeding, perforation or displacement after BD stent implantation.
		BM 4		Expectoration of small stent particles		Four patients needed multiple stenting for anastomotic re-stenosis.
14	50	BS^†^	A	Stent fracture	26%	Reduced pulmonary infection rates for up to 1 year after their insertion.
		BM^†^		Liatrogenically damaged		Improvements in lung function
				Collapse		
				Obstruction		
15	28	BS 23	Right main bronchus 15 Bronchus intermedius 2	Granulomas	26%	Immediate symptomatic and functional improvement.
		BM 5	NA	Partial fibrosis stenosis		Improved FEV_1_, FVC, FEF50%
				Hemoptysis		
				Stent broke		
16	11	BS 3	Right main bronchus + Bronchus intermedius 3	Granulation	10%	Functional improvement was immediate with a 35% mean FEV_1_ gain
		BM 3	NA			Previous recurrent episodes of pneumonia symptoms did not occur again
		BS + BM 5	Right main bronchus 5			
17	11	BS 11	Right anastomosis 3	Infection	33.40%	All patients had increased lung function
				Colonization		
				Accumulation of secretions		

NA, not available; BOS, bronchiolitis obliterans syndrome; DAS, distal airway strictures; CAS, central airway strictures; BS, bronchial stenosis; BM, bronchomalacia; AD, anastomotic dehiscence.

^*^The number of stents for BS or AD respectively is unavailable.

^†^The number of stents for BS or BM respectively is unavailable.

Almost all studies indicated that stent insertion could immediately palliate stenosis symptoms such as dyspnoea, improve lung function (11, 13–16, 19–21, 23–26), decrease the degree of stenosis (23) and successfully re-open the airways (16). Regarding long-term efficacy, most studies indicated that the survival time of post-stent patients was similar to that of patients without stents (3, 11, 20, 24, 25). However, Gottlieb et al. (16) reported significantly lower survival rate in LTx recipients with stents than in the total LTx cohort. The overall survival analysis was only synthesized with seven of included studies. The median overall survival time was 1,124 (95% confidence interval 415–1,833) days in stented patients (Supplementary Figure 1, Supplementary Table 1). Concerning permanent stent removal, three studies (11, 20, 26) suggested that stent placement seemed to result in a permanent resolution of airway stenosis. Dutau et al. (11), Thistlethwaite et al. (20), and Sundset et al. (26) showed that 80 (16/20), 69.5 (16/23), and 81.5% (22/27) of patients in their studies obtained permanent stent removal without airway stenosis recurrence, respectively.

In addition, the number of bronchoscopies was also recorded in the follow-up. DiBardino et al. (7) indicated that no significant reduction in bronchoscopies per month was observed in stented patients compared to those never stented; meanwhile, both groups underwent DAS with different types. Ma et al. (21) found a statistically significant reduction in the number of bronchoscopies per month after stent removal compared to pre-stent placement for patients who ultimately could undergo stent removal. Regarding long-term lung function improvement for more than 3 months, some studies demonstrated that FEV_1_ improved significantly after stent implantation (11, 14, 19, 21, 24, 26). However, Izhakian et al. (24) suggested that FEV_1_ values were significantly lower in patients with stents than those without stents at 2.5 years post-transplantation but did not differ significantly between the two groups at 5, 7.5, and 10 years. Baljendra et al. (19) showed that the statistically significant improvement in the FEV_1_ value after stent implantation was transient, was sustained at 6 months post-stent implantation (19), and then (12 months) the changes in FEV_1_ may return to baseline. A study by Burns et al. (14) discovered that the infection rate decreased significantly from the 12-month preceding stent implantation to the corresponding period after implantation.

The safety of stent therapy was considered relatively good, although some common complications of stent migration, granulation tissue formation and mucus plugging were only mild to moderate and could be resolved through intervention (11). Moreover, the risk of stent-related complications does not seem to be associated with different types of stents (21, 23). However, some studies have suggested that silicone stents can avoid several complications of metal stents, such as stent fracture, and can be removed after lesion maturity (20, 26). Novel materials, such as biodegradable stents (13) also demonstrated good efficacy and safety.

### Assessment of study quality

All the seven case-series studies were rated as high quality, with three of them attained seven “Yes” response and four attained eight “Yes” response (Supplementary Table 2). For remaining cohort studies, eight studies were assessed as high-quality studies and two were rated as moderate quality (Supplementary Table 3).

## Discussion

Although the first human lung transplantation was carried out was similar to other organ transplants, it experienced a longer development. The reasons hindering the maturation of the technology are mainly related to the healing problem of bronchial anastomosis, high immunogenicity of the lungs, and the risk of infection (30). Airway complications, particularly bronchial stenosis, still affect 7–40% of lung transplant patients (31). Flexible bronchoscopy and balloon dilation are commonly used in the treatment of airway stenosis, and various silicone and expandable metal stents have been used (12). However, the available studies were all retrospective with a limited sample size.

Almost all the 17 studies enrolled indicated that patients experienced immediate symptom relief and improved lung function. However, the follow-up results were controversial despite the majority of researchers identifying the long-term efficacy of stent therapy for post-transplantation airway stenosis (3, 11, 14, 20, 24–26). It cannot be ignored that some authors considered the improvement unsustainable. When comparing patients stented and never stented (7) and pre- and post-stent (19), lung function returned to baseline after 12 months (19), or only in patients who ultimately were able to undergo stent removal could improvement be observed (20). The limited sample size might be part of the reason because the referred studies were retrospective studies with sample sizes of 4 (32), 20 (20), and 25 (19) respectively. In addition, most studies (12/17) lacked a control group.

Studies recorded patients developing bronchiolitis obliterans syndrome (BOS) and died after stent insertion (16, 28) or after stent removal (26). Interestingly, in study of Gottlieb et al. (16), the overall survival time of patients undergoing stent insertion was significantly lower than the whole LTx cohort, while the BOS-freedom survival time was not affected. This might suggest BOS an additional factor to influence the long-term survival time of LTx patients, and stent-therapy may have limited potenty to alleviate the LTx complication in patients concurrent with BOS, and thus performing little improvement in the long-term survival rate. However, in regard of lacking enough survival data of subgroup of patients with BOS, we were not capable to draw a convincing conclusion about whether long-term survival efficacy of stent insertion could be influenced by existed or developing BOS or not. Moreover, only 7 studies were available to estimate overall survival rate of stented patients, without patients with airway complications and without stents as controlled group. The biases from limited reported case could not be ignored as well.

For the survival rate, the previous studies provided the limited ability to draw an exact conclusion of the long-term efficacy of stent insertion on survival outcome, due to the potential reason of the control group. In those retrospective cohorts or case series, patients undergoing stent insertion might be more likely to have worse clinical manifestations, such as worse symptoms, poorer lung function, and severer stenosis, than those who did not need stent insertion with or without airway stenosis. Additionally, patients were required to acute stent insertion to save their lives when airway complications led to acute respiratory distress in clinical practice. They would be included as interventional groups in retrospective studies. Thus, patients with airway stenosis and no airway stent were performed as better clinical manifestations and very few. This might be why the control group in previous studies were often those without airway stenosis or the whole LTx cohort, with better baseline conditions. In this situation, comparing survival time between the intervention group and the control group might underestimate the long-term efficacy of stent in LTx patients with airway complications. In terms of clinical practice, conducting a randomized control trial to verify this conclusion would also be challenging. Therefore, evaluating the long-term survival effect of stent insertion relying on the present studies was difficult.

DiBardino et al. (7) and Ma et al. (21) drew different conclusions regarding the reduction in the number of bronchoscopies after stent insertion. This might be partly explained by the heterogeneity between two studies. We noticed an obvious difference in the population between the two studies (21, 32). DiBardino et al. (7) conducted their study among patients who were stented or non-stented. In contrast, Ma et al. (21) compared data between patients who ultimately underwent stent removal and those with an indefinite stent period. First, the patients needing a stent differ from those who do not need one. Stent therapy tends to be chosen for those who cannot benefit from conventional treatment, such as balloon dilation, or those who undergo high-level obstruction and recur fast. Therefore, these patients are bound to undergo frequent bronchoscopies and stent replacement (33) and thus have poorer follow-up results. In addition, the individuals were also highly heterogeneous because of the different endotypes of DAC based on the bronchoscopic appearance. DiBardino et al. (7) indicated that although the mechanism was elusive, patients may respond differently to stenting due to the different linked biology of each endotype. Patient with the endotype of granulation tissue may be exacerbated by the insertion of stents, while patient with cicatrix scar endotype of stenosis respond well to stents (7). Therefore, to evaluate whether stent therapy is effective and safe for the treatment of airway stenosis after LTx, some potential factors involved are of sustained concern.

### Stenosis type

Bronchial stenosis can be roughly classified

Bronchial stenosis can be roughly classified into two types: stenosis localized at or within 1.0 cm of the suture line is called CAS, while stenosis localized distally to the anastomotic stoma or lobar bronchus is called DAS, with or without CAS. DAS is referred to as vanishing bronchus intermedius syndrome, affecting the intermediate bronchus or lobar airways, with a median survival of only 25 months (10). Early postoperative airway stenosis is usually associated with oedema and necrosis of the mucosa, while late stenosis is mainly due to bronchomalacia or bronchofibrosis caused by airway ischemia, recurrent infection, or rejections (34), with a clinical incidence of about 13 (35) and 2.5–3% (10), respectively.

Besides, Thistlethwaite et al. (20) reported that pulmonary aspergillosis and pseudomonal infection, age <45 years, and early rejection were correlated with airway stenosis. They also reported no obvious association with the underlying lung disease; however, only a small sample size was included. One study (7) indicated that some DAS endotypes, of which the bronchoscopic appearance was cicatrix scar, seemed to respond favorably to airway stenting and may be related to biological repair mechanisms. Another study (21) also indicated that endoscopic phenotypes and the anatomy of airway stenosis are major factors associated with stent success. Nevertheless, these results suggest that the stenosis type is a non-negligible factor in analyzing the efficacy of stenting.

### Stent materials

Some studies have suggested that no matter what criteria the stent selection is based on, the risk of stent-related complications does not seem to differ with different stent types (21, 23). Of the 17 studies enrolled, three studies that deployed silicone stents reported that patients could receive permanent benefits from silicone stent insertion without recurrence during follow-up. The silicone stents studied were thin-walled and expandable, which, to some extent, avoided the disadvantages of traditional silicone stents, despite their shortcomings. The stents could be removed 10–12 months after placement in most patients, and the improvement in lung function was maintained after removal (20). New stent materials have also been applied in esophageal, intestinal, urethral, bile duct, and vascular stenosis (13). The biodegradable polydioxanone stents could be degraded into harmless degradation products in the body, the process of which were accelerated under low pH conditions (36). In the application process for patients with bronchial stenosis, biodegradable stents can be customized and easily handled, but there are still inevitable complications. Some patients have been reported to develop sputum with small stent particles, while it was not dangerous. They may also induce growth of granulations that led to restenosis (13, 36). The predominance of novel stents still requires a long-term case-control study with a large sample size. Therefore, the effects of stent selection on stent complications, stent effectiveness, and patient survival rates need to be verified in more large-scale control studies.

### Suture technique

There are roughly four types of lung transplantation: single lung transplantation, double lung transplantation, heart-lung transplantation, and living lung lobar transplantation. According to the number of dangerous anastomoses, some studies found that these complications were significantly higher in SLT recipients than in DLT recipients (12). However, it seems that the lung transplantation type cannot be changed, while the suture technique is the most important factor for effective anastomotic healing, which can be continuously improved. In lung transplantation, continuous sutures are used for the membrane. In contrast, there are various suture methods for cartilage: simple continuous suture, simple discontinuous suture, eight-figure discontinuous suture, horizontal mattress + eight-figure discontinuous suture, telescopic suture, and telescopic suture (bedding suture) (37). The telescoping anastomotic technique has been observed an association with high rate of stenosis (38, 39) and considered as a risk factor to develop airway complications (40), while continuous end-to-end suture technology with absorbable sutures can provide a satisfactory effect and significantly reduce the incidence of airway complications including stenosis (40, 41). Appropriate suture techniques can improve the ischaemia of donor bronchi and reduce the risk of bronchial stenosis (31), which might indicate the different repair mechanism behind different suture techniques, and thus lead to diverse endotypes of stenosis.

This study has the following limitations: First, limited cases of LTx patients restricted the sample size of studies to evaluate the efficacy and safety of stents in lung-transplantation patients with airway stenosis. Second, Airway stenosis seriously affect the clinical outcomes and long-term prognosis of lung-transplantation patients and thus airway stenting provides effective palliation for patients with airway stenosis, both of which limits the feasibility of controlled studies with considerable sample size, meanwhile, limited our ability to evaluate the exact long-term efficacy of stent insertion on survival outcomes in LTx patients with airway complications. Moreover, the intervention implemented to patients vary a lot among studies, due to the inconsistent procedure of managing airway stenosis. The factors above consist of the heterogeneity among the included studies, which makes it difficult to conduct statistical synthesis. Hence, studies with appropriate sample size and integrated demographics of patients, aiming at the bronchoscopic interventions for the airway stenosis after lung transplantation, are desirable in future exploration due to the lack of the sound evidence.

### Points for clinical practice

Regarding the ethical concern and the difficulty to match a control group in evaluating the long-term efficacy and safety of stents in airway stenosis, the future effort could be focused on the prospective studies with large-scale sample, exploring the baseline characteristics of LTx patients with airway stenosis as predictors to the good therapy response to stent insertion, such as the primary diseases leading the lung transplantation, endobronchial phenotypes of stenosis, the suture technique of lung transplantation. The evaluation of efficacy of stent therapy is suggested to conducted in the subgroups of patients who performed as different stenosis types (DAS, CAS) and who are concurrent with or developing BOS after LTx. This might help to identify the population that may not be able to response well to stent insertion. Identify the specific effect of a certain type of stent on a subset of patients and provide refined conclusions. In addition, little attention had been paid to the timing of stent implantation and the way of stent deployment. Novel biodegradable stent, in this situation, is worth to pay more attention on its customized and easily-handle features.

In summary, endobronchial stent implantation is a relatively safe and effective method for the treatment of airway stenosis after lung transplantation, especially regarding the short-term efficacy of symptom relief and improvement in pulmonary function. However, the long-term efficacy on lung function is not distinct and appears to require more frequent bronchoscopies, the efficacy of stent insertion on survival outcome needs further investigation; Stent-related complications are common regardless of the material used. However, serious complications are rare and can be improved with routine management. In addition to continuously optimizing transplantation techniques and strengthening the management of donor recipients during the perioperative period, it is also necessary to strictly evaluate potential beneficiaries and select appropriate stent materials to further improve the long-term outcomes.

## Data availability statement

The original contributions presented in the study are included in the article/Supplementary material, further inquiries can be directed to the corresponding author.

## Author contributions

Conception and design: GH. Collection and assembly of data: ZW, BZ, and YB. Data analysis and interpretation: MD, RT, and QZ. Manuscript writing and final approval of manuscript: All authors.
