# Supplementary material for: Utility and safety of airway stenting in airway stenosis after lung transplant: A systematic review
Source: Front Med (Lausanne). 2023 Mar 9;10:1061447. doi: 10.3389/fmed.2023.1061447 (PMC10034355; doi:10.3389/fmed.2023.1061447)
Supplement: Supplementary file 1 [file Data_Sheet_1.docx]

**Search strategy**

**PubMed = 126**

| **Search number** | **Query** | **Sort By** | **Filters** | **Results** | **Time** |
| --- | --- | --- | --- | --- | --- |
| **19** | (("Lung Transplantation"[Mesh]) OR (Grafting, Lung[Title/Abstract]) OR (Graftings, Lung[Title/Abstract]) OR (Lung Grafting[Title/Abstract]) OR (Lung Graftings[Title/Abstract]) OR (Transplantation, Lung[Title/Abstract]) OR (Lung Transplantations[Title/Abstract]) OR (Transplantations, Lung[Title/Abstract])) AND ("airway"[Title/Abstract] OR "airways"[Title/Abstract]) AND (("Constriction, Pathologic"[Mesh]) OR (Constrictions, Pathologic[Title/Abstract]) OR (Pathologic Constrictions[Title/Abstract]) OR (Stricture[Title/Abstract]) OR (Strictures[Title/Abstract]) OR (Stenosis[Title/Abstract]) OR (Stenoses[Title/Abstract]) OR (Constriction, Pathological[Title/Abstract]) OR (Pathological Constriction[Title/Abstract]) OR (Pathologic Constriction[Title/Abstract]) OR ("complications" [Subheading]) OR (associated disease[Title/Abstract]) OR (sequelae[Title/Abstract]) OR (sequels[Title/Abstract]) OR (coexistent disease[Title/Abstract]) OR (concomitant disease[Title/Abstract]) OR (associated conditions[Title/Abstract]) OR (coexistent conditions[Title/Abstract]) OR (concomitant conditions[Title/Abstract])) AND ((stent[Title/Abstract]) OR (stents[Title/Abstract]) OR ("Stents"[Mesh])) |  |  | 126 | 09:51:18 |
| **17** | ((stent[Title/Abstract]) OR (stents[Title/Abstract])) OR ("Stents"[Mesh]) |  |  | 122,583 | 09:37:22 |
| **16** | (stent[Title/Abstract]) OR (stents[Title/Abstract]) |  |  | 97,828 | 09:36:06 |
| **13** | ("Constriction, Pathologic"[Mesh]) OR (Constrictions, Pathologic[Title/Abstract]) OR (Pathologic Constrictions[Title/Abstract]) OR (Stricture[Title/Abstract]) OR (Strictures[Title/Abstract]) OR (Stenosis[Title/Abstract]) OR (Stenoses[Title/Abstract]) OR (Constriction, Pathological[Title/Abstract]) OR (Pathological Constriction[Title/Abstract]) OR (Pathologic Constriction[Title/Abstract]) OR ("complications" [Subheading]) OR (associated disease[Title/Abstract]) OR (sequelae[Title/Abstract]) OR (sequels[Title/Abstract]) OR (coexistent disease[Title/Abstract]) OR (concomitant disease[Title/Abstract]) OR (associated conditions[Title/Abstract]) OR (coexistent conditions[Title/Abstract]) OR (concomitant conditions[Title/Abstract]) |  |  | 2,547,568 | 09:34:41 |
| **12** | "airway"[Title/Abstract] OR "airways"[Title/Abstract] |  |  | 182,552 | 09:33:38 |
| **10** | ("Constriction, Pathologic"[Mesh]) OR ((Constrictions, Pathologic[Title/Abstract]) OR (Pathologic Constrictions[Title/Abstract]) OR (Stricture[Title/Abstract]) OR (Strictures[Title/Abstract]) OR (Stenosis[Title/Abstract]) OR (Stenoses[Title/Abstract]) OR (Constriction, Pathological[Title/Abstract]) OR (Pathological Constriction[Title/Abstract]) OR (Pathologic Constriction[Title/Abstract])) |  |  | 219,839 | 09:29:39 |
| **9** | (Constrictions, Pathologic[Title/Abstract]) OR (Pathologic Constrictions[Title/Abstract]) OR (Stricture[Title/Abstract]) OR (Strictures[Title/Abstract]) OR (Stenosis[Title/Abstract]) OR (Stenoses[Title/Abstract]) OR (Constriction, Pathological[Title/Abstract]) OR (Pathological Constriction[Title/Abstract]) OR (Pathologic Constriction[Title/Abstract]) |  |  | 208,434 | 09:28:22 |
| **8** | ("complications" [Subheading]) OR ((associated disease[Title/Abstract]) OR (sequelae[Title/Abstract]) OR (sequels[Title/Abstract]) OR (coexistent disease[Title/Abstract]) OR (concomitant disease[Title/Abstract]) OR (associated conditions[Title/Abstract]) OR (coexistent conditions[Title/Abstract]) OR (concomitant conditions[Title/Abstract])) |  |  | 2,377,253 | 09:27:47 |
| **7** | (associated disease[Title/Abstract]) OR (sequelae[Title/Abstract]) OR (sequels[Title/Abstract]) OR (coexistent disease[Title/Abstract]) OR (concomitant disease[Title/Abstract]) OR (associated conditions[Title/Abstract]) OR (coexistent conditions[Title/Abstract]) OR (concomitant conditions[Title/Abstract]) |  |  | 85,905 | 09:27:24 |
| **6** | ("Lung Transplantation"[Mesh]) OR ((Grafting, Lung[Title/Abstract]) OR (Graftings, Lung[Title/Abstract]) OR (Lung Grafting[Title/Abstract]) OR (Lung Graftings[Title/Abstract]) OR (Transplantation, Lung[Title/Abstract]) OR (Lung Transplantations[Title/Abstract]) OR (Transplantations, Lung[Title/Abstract])) |  |  | 45,631 | 09:26:49 |
| **5** | (Grafting, Lung[Title/Abstract]) OR (Graftings, Lung[Title/Abstract]) OR (Lung Grafting[Title/Abstract]) OR (Lung Graftings[Title/Abstract]) OR (Transplantation, Lung[Title/Abstract]) OR (Lung Transplantations[Title/Abstract]) OR (Transplantations, Lung[Title/Abstract]) |  |  | 43,464 | 09:26:26 |
| **4** | "Stents"[Mesh] | Most Recent |  | 87,242 | 09:25:50 |
| **3** | "Constriction, Pathologic"[Mesh] | Most Recent |  | 33,761 | 09:24:53 |
| **2** | "complications" [Subheading] | Most Recent |  | 2,313,994 | 09:24:34 |
| **1** | "Lung Transplantation"[Mesh] | Most Recent |  | 18,216 | 09:23:37 |

**Embase = 257**

| **No.** | **Query** | **Results** | **Date** |
| --- | --- | --- | --- |
| **#6** | #2 AND #3 AND #4 AND #5 | 257 | 12 Sep 2022 |
| **#5** | 'stent'/exp OR stents:ti,ab OR stent:ti,ab | 229543 | 12 Sep 2022 |
| **#4** | 'airway'/exp OR airway:ti,ab OR airways:ti,ab | 265470 | 12 Sep 2022 |
| **#3** | 'stenosis, occlusion and obstruction'/exp OR stenosis:ti,ab OR 'complication'/exp OR complication:ti,ab | 2424383 | 12 Sep 2022 |
| **#2** | 'lung transplantation'/exp OR 'lung transplantation':ti,ab | 44873 | 12 Sep 2022 |
| **#1** | 'stenosis, occlusion and obstruction'/exp OR 'stenosis, occlusion and obstruction' OR stenosis:ti,ab OR complication:ti,ab OR 'complication'/exp OR 'complication' | 3846802 | 12 Sep 2022 |

**Cochrane library**

Search Name: LTx cochrane

Date Run: 12/09/2022 05:42:42

Comment:

ID Search Hits

#1 MeSH descriptor: [Lung Transplantation] explode all trees 241

#2 (Lung Grafting or Transplantations, Lung or Graftings, Lung or Lung Transplantations or Grafting, Lung or Transplantation, Lung or Lung Graftings):ti,ab,kw 2321

#3 #1 or #2 2321

#4 MeSH descriptor: [Constriction, Pathologic] explode all trees 915

#5 (Stricture or Constrictions, Pathologic or Stenoses or Constriction, Pathological or Stenosis or Strictures or Pathologic Constrictions or Pathologic Constriction or Pathological Constriction):ti,ab,kw 15776

#6 #4 or #5 15776

#7 MeSH descriptor: [Postoperative Complications] explode all trees 43864

#8 (Complications, Postoperative or Complication, Postoperative or Postoperative Complication):ti,ab,kw 60346

#9 #7 or #8 80129

#10 #6 or #9 93687

#11 (airway or airways):ti,ab,kw 28461

#12 #11 and #10 1861

#13 MeSH descriptor: [Stents] explode all trees 4571

#14 (stent or stents):ti,ab,kw 15776

#15 #13 or #14 15776

#16 #3 and #12 and #15 2
