# Supplementary material for: Utility and safety of airway stenting in airway stenosis after lung transplant: A systematic review
Source: Front Med (Lausanne). 2023 Mar 9;10:1061447. doi: 10.3389/fmed.2023.1061447 (PMC10034355; doi:10.3389/fmed.2023.1061447)
Supplement: Supplementary file 2 [file Image_1.pdf]

Supplementary figure 1

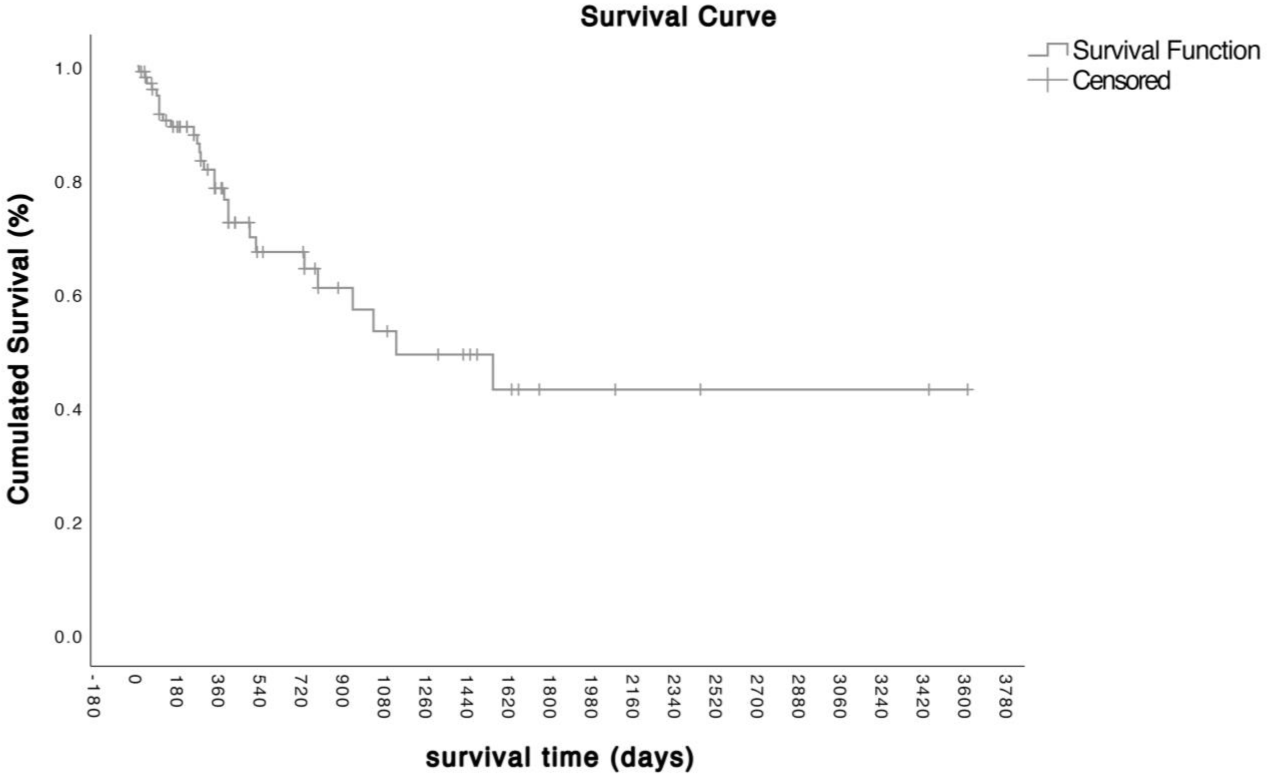

Supplementary figure 1. Kaplan-Meier estimate survival curve in patients undergoing stent insertion among the seven studies. The median of overall survival time is 1124 (95% confidence interval 415 to 1833) days.
