# Supplementary material for: Utility and safety of airway stenting in airway stenosis after lung transplant: A systematic review
Source: Front Med (Lausanne). 2023 Mar 9;10:1061447. doi: 10.3389/fmed.2023.1061447 (PMC10034355; doi:10.3389/fmed.2023.1061447)
Supplement: Supplementary file 3 [file Table_1.docx]

Supplementary Tables

**Supplementary Table 1.**

Supplementary Table 1. Survival table in patients undergoing stent insertion among the seven studies.

| Number | Time | Status | Cumulative Proportion Surviving at the Time | | Number of Cumulative Events | Number of Remaining Cases |
| --- | --- | --- | --- | --- | --- | --- |
|  |  |  | Estimate | Std. Error |  |  |
| 1 | 7 | 0 | 0.99 | 0.01 | 1 | 101 |
| 2 | 17 | 1 | . | . | 1 | 100 |
| 3 | 31 | 1 | . | . | 1 | 99 |
| 4 | 35 | 0 | 0.98 | 0.014 | 2 | 98 |
| 5 | 35 | 1 | . | . | 2 | 97 |
| 6 | 35 | 1 | . | . | 2 | 96 |
| 7 | 35 | 1 | . | . | 2 | 95 |
| 8 | 35 | 1 | . | . | 2 | 94 |
| 9 | 35 | 1 | . | . | 2 | 93 |
| 10 | 41 | 0 | 0.97 | 0.017 | 3 | 92 |
| 11 | 62 | 1 | . | . | 3 | 91 |
| 12 | 63 | 0 | 0.959 | 0.02 | 4 | 90 |
| 13 | 65 | 1 | . | . | 4 | 89 |
| 14 | 65 | 1 | . | . | 4 | 88 |
| 15 | 84 | 0 | 0.948 | 0.023 | 5 | 87 |
| 16 | 95 | 0 | . | . | 6 | 86 |
| 17 | 95 | 0 | . | . | 7 | 85 |
| 18 | 95 | 0 | 0.915 | 0.029 | 8 | 84 |
| 19 | 95 | 1 | . | . | 8 | 83 |
| 20 | 111 | 0 | 0.904 | 0.03 | 9 | 82 |
| 21 | 125 | 1 | . | . | 9 | 81 |
| 22 | 146 | 0 | 0.893 | 0.032 | 10 | 80 |
| 23 | 155 | 1 | . | . | 10 | 79 |
| 24 | 155 | 1 | . | . | 10 | 78 |
| 25 | 173 | 1 | . | . | 10 | 77 |
| 26 | 175 | 1 | . | . | 10 | 76 |
| 27 | 185 | 1 | . | . | 10 | 75 |
| 28 | 185 | 1 | . | . | 10 | 74 |
| 29 | 185 | 1 | . | . | 10 | 73 |
| 30 | 185 | 1 | . | . | 10 | 72 |
| 31 | 185 | 1 | . | . | 10 | 71 |
| 32 | 185 | 1 | . | . | 10 | 70 |
| 33 | 185 | 1 | . | . | 10 | 69 |
| 34 | 185 | 1 | . | . | 10 | 68 |
| 35 | 185 | 1 | . | . | 10 | 67 |
| 36 | 185 | 1 | . | . | 10 | 66 |
| 37 | 215 | 1 | . | . | 10 | 65 |
| 38 | 215 | 1 | . | . | 10 | 64 |
| 39 | 215 | 1 | . | . | 10 | 63 |
| 40 | 215 | 1 | . | . | 10 | 62 |
| 41 | 245 | 0 | 0.879 | 0.035 | 11 | 61 |
| 42 | 245 | 1 | . | . | 11 | 60 |
| 43 | 245 | 1 | . | . | 11 | 59 |
| 44 | 245 | 1 | . | . | 11 | 58 |
| 45 | 260 | 0 | 0.864 | 0.037 | 12 | 57 |
| 46 | 270 | 0 | 0.849 | 0.039 | 13 | 56 |
| 47 | 275 | 0 | 0.833 | 0.042 | 14 | 55 |
| 48 | 275 | 1 | . | . | 14 | 54 |
| 49 | 289 | 0 | 0.818 | 0.044 | 15 | 53 |
| 50 | 305 | 1 | . | . | 15 | 52 |
| 51 | 305 | 1 | . | . | 15 | 51 |
| 52 | 305 | 1 | . | . | 15 | 50 |
| 53 | 335 | 0 | . | . | 16 | 49 |
| 54 | 335 | 0 | 0.785 | 0.048 | 17 | 48 |
| 55 | 335 | 1 | . | . | 17 | 47 |
| 56 | 339 | 1 | . | . | 17 | 46 |
| 57 | 365 | 1 | . | . | 17 | 45 |
| 58 | 365 | 1 | . | . | 17 | 44 |
| 59 | 365 | 1 | . | . | 17 | 43 |
| 60 | 365 | 1 | . | . | 17 | 42 |
| 61 | 365 | 1 | . | . | 17 | 41 |
| 62 | 365 | 1 | . | . | 17 | 40 |
| 63 | 369 | 1 | . | . | 17 | 39 |
| 64 | 377 | 0 | 0.765 | 0.05 | 18 | 38 |
| 65 | 395 | 0 | . | . | 19 | 37 |
| 66 | 395 | 0 | 0.725 | 0.055 | 20 | 36 |
| 67 | 395 | 1 | . | . | 20 | 35 |
| 68 | 395 | 1 | . | . | 20 | 34 |
| 69 | 396 | 1 | . | . | 20 | 33 |
| 70 | 423 | 1 | . | . | 20 | 32 |
| 71 | 425 | 1 | . | . | 20 | 31 |
| 72 | 425 | 1 | . | . | 20 | 30 |
| 73 | 485 | 1 | . | . | 20 | 29 |
| 74 | 485 | 1 | . | . | 20 | 28 |
| 75 | 488 | 0 | 0.699 | 0.059 | 21 | 27 |
| 76 | 515 | 0 | 0.673 | 0.062 | 22 | 26 |
| 77 | 520 | 1 | . | . | 22 | 25 |
| 78 | 545 | 1 | . | . | 22 | 24 |
| 79 | 720 | 1 | . | . | 22 | 23 |
| 80 | 725 | 0 | 0.644 | 0.066 | 23 | 22 |
| 81 | 725 | 1 | . | . | 23 | 21 |
| 82 | 725 | 1 | . | . | 23 | 20 |
| 83 | 771 | 1 | . | . | 23 | 19 |
| 84 | 784 | 0 | 0.61 | 0.071 | 24 | 18 |
| 85 | 785 | 1 | . | . | 24 | 17 |
| 86 | 872 | 1 | . | . | 24 | 16 |
| 87 | 935 | 0 | 0.572 | 0.076 | 25 | 15 |
| 88 | 1025 | 0 | 0.534 | 0.08 | 26 | 14 |
| 89 | 1085 | 1 | . | . | 26 | 13 |
| 90 | 1124 | 0 | 0.493 | 0.084 | 27 | 12 |
| 91 | 1306 | 1 | . | . | 27 | 11 |
| 92 | 1415 | 1 | . | . | 27 | 10 |
| 93 | 1445 | 1 | . | . | 27 | 9 |
| 94 | 1475 | 1 | . | . | 27 | 8 |
| 95 | 1544 | 0 | 0.431 | 0.093 | 28 | 7 |
| 96 | 1625 | 1 | . | . | 28 | 6 |
| 97 | 1655 | 1 | . | . | 28 | 5 |
| 98 | 1745 | 1 | . | . | 28 | 4 |
| 99 | 2075 | 1 | . | . | 28 | 3 |
| 100 | 2445 | 1 | . | . | 28 | 2 |
| 101 | 3437 | 1 | . | . | 28 | 1 |
| 102 | 3605 | 1 | . | . | 28 | 0 |

Status = 0 means dead status, status = 1 means alive status.

Supplementary Table 2. The Joanna Briggs Institute (JBI) Quality Assessment for Case series

| **Study** | **Question 1** | **Question 2** | **Question 3** | **Question 4** | **Question 5** | **Question 6** | **Question 7** | **Question 8** | **Result** | **Quality assessment** |
| --- | --- | --- | --- | --- | --- | --- | --- | --- | --- | --- |
| Kapoor, et al | **Yes** | **Yes** | **Yes** | **Yes** | **Yes** | **Yes** | **Yes** | **Yes** | **8** | **high** |
| Fernandez-Bussy, et al | **Yes** | **No** | **Yes** | **Yes** | **Yes** | **Yes** | **Yes** | **Yes** | **7** | **high** |
| Sundset, et al | **Yes** | **No** | **Yes** | **Yes** | **Yes** | **Yes** | **Yes** | **Yes** | **7** | **high** |
| Susanto, et al | **Yes** | **Yes** | **Yes** | **Yes** | **Yes** | **Yes** | **Yes** | **Yes** | **8** | **high** |
| Lischke, et al | **Yes** | **Yes** | **Yes** | **Yes** | **Yes** | **Yes** | **Yes** | **Yes** | **8** | **high** |
| Bolot, et al | **Yes** | **Yes** | **Yes** | **Yes** | **Yes** | **Yes** | **Yes** | **Yes** | **8** | **high** |
| Anile, et al | **Yes** | **No** | **Yes** | **Yes** | **Yes** | **Yes** | **Yes** | **Yes** | **7** | **high** |

**Question 1:** Were patient’s demographic characteristics clearly described? **Question 2:** Was the patient’s history clearly described and presented as a timeline? **Question 3:** Was the current clinical condition of the patient on presentation clearly described? **Question 4:** Were diagnostic tests or assessment methods and the results clearly described? **Question 5:** Was the intervention(s) or treatment procedure(s) clearly described? **Question 6:** Was the post-intervention clinical condition clearly described? **Question 7:** Were adverse events (harms) or unanticipated events identified and described? **Question 8:** Does the case report provide takeaway lessons?

high quality: 7-8 “Yes” responses, moderate quality: 4-6 “Yes” responses, low quality: 1-3 “Yes” responses.

**Supplementary Table 3. The Joanna Briggs Institute (JBI) Critical Quality Assessment for Cohort Study**

| **Study** | **Question 1** | **Question 2** | **Question 3** | **Question 4** | **Question 5** | **Question 6** | **Question 7** | **Question 8** | **Question 9** | **Question 10** | **Question 11** | **Results** | **Quality assessment** |
| --- | --- | --- | --- | --- | --- | --- | --- | --- | --- | --- | --- | --- | --- |
| DiBardino, et al | **No** | **Yes** | **Yes** | **No** | **No** | **Yes** | **Yes** | **Yes** | **Yes** | **Yes** | **Yes** | **8** | **high** |
| Thistlethwaite, et al | **No** | **Yes** | **Yes** | **No** | **No** | **Yes** | **Yes** | **Yes** | **Yes** | **Yes** | **Yes** | **8** | **high** |
| Ma, et al | **No** | **No** | **Yes** | **Yes** | **No** | **Yes** | **Yes** | **Yes** | **Yes** | **Yes** | **Yes** | **8** | **high** |
| Dutau, et al | **No** | **Yes** | **Yes** | **No** | **No** | **Yes** | **Yes** | **Yes** | **Yes** | **Yes** | **Yes** | **8** | **high** |
| Izhakian, et al | **Yes** | **Yes** | **Yes** | **Yes** | **Yes** | **Yes** | **Yes** | **Yes** | **Yes** | **Yes** | **Yes** | **11** | **high** |
| Abdel-Rahman, et al | **No** | **Yes** | **Yes** | **No** | **No** | **Yes** | **Yes** | **Yes** | **Yes** | **Yes** | **Yes** | **8** | **high** |
| Saad, et al | **No** | **Yes** | **Yes** | **No** | **No** | **Yes** | **Yes** | **Yes** | **Yes** | **Yes** | **Yes** | **8** | **high** |
| Gottlieb, et al | **No** | **No** | **Yes** | **No** | **No** | **Yes** | **Yes** | **Yes** | **Yes** | **Yes** | **Yes** | **7** | **moderate** |
| Burns, et al | **No** | **No** | **Yes** | **Yes** | **Yes** | **Yes** | **Yes** | **Yes** | **Yes** | **Yes** | **Yes** | **9** | **high** |
| Fonseca, et al | **No** | **No** | **Yes** | **No** | **No** | **Yes** | **Yes** | **Yes** | **Yes** | **Yes** | **Yes** | **6** | **moderate** |

**Question 1：**Were the two groups similar and recruited from the same population? **Question 2：**Were the exposures measured similarly to assign people to both exposed and unexposed groups? **Question 3：**Was the exposure measured in a valid and reliable way? **Question 4：**Were confounding factors identified? **Question 5:** Were strategies to deal with confounding factors stated? **Question 6:** Were the groups/participants free of the outcome at the start of the study (or at the moment of exposure)? **Question 7:** Were the outcomes measured in a valid and reliable way? **Question 8:** Was the follow up time reported and sufficient to be long enough for outcomes to occur? **Question 9:** Was follow up complete, and if not, were the reasons to loss to follow up described and explored? **Question 10:** Were strategies to address incomplete follow up utilized? **Question 11:** Was appropriate statistical analysis used?

high quality: 8-11 “Yes” responses, moderate quality: 4-7 “Yes” responses, low quality: 1-3 “Yes” response
